# Relationship between eating disorders perception and psychosocial profile in school-dropout adolescents

**DOI:** 10.1186/s40337-023-00783-2

**Published:** 2023-04-11

**Authors:** Francesca Mastorci, Maria Francesca Lodovica Lazzeri, Paolo Piaggi, Cristina Doveri, Anselmo Casu, Gabriele Trivellini, Irene Marinaro, Cristina Vassalle, Alessandro Pingitore

**Affiliations:** 1 Clinical Physiology Institute, CNR, Via Moruzzi, 1, 56124 Pisa, Italy; 2 Department of Information Engineering, University of Pisa, Pisa, Italy; 3 Fondazione Toscana Gabriele Monasterio, Pisa, Italy

**Keywords:** Health-related quality of life, Adolescence, Eating disorders, Body perception, Well-being

## Abstract

**Background:**

In body-mind relationship field, eating disorders (ED) are considered disabling disorders that can alter physical health status, inducing profound alterations in psychosocial, cognitive and emotional dimensions. These disorders, characterized by a strong comorbidity with other diseases, usually begin during childhood or adolescence, and include anorexia nervosa, bulimia nervosa and binge eating. Aim of this study was to investigate the associations between eating disorders perception and dimensions of health-related quality of life (HRQoL) and well-being perception (WBP) in school-dropout adolescents.

**Methods:**

Data were collected in 450 adolescents (19 ± 2 years, male 308), and HRQoL, WBP, and ED were assessed by means a battery of standardized questionnaire.

**Results:**

EDs are more pronounced in females than in males (*p* < 0.05) and are associated with lower HRQoL (*p* < 0.001) and lower well-being perception (*p* < 0.001). EDs are associated with an impairment of physical (*p* < 0.05) and psychological well-being perception (*p* < 0.001), emotional responses (*p* < 0.001), self-perception (*p* < 0.001), and a reduction of general well-being (*p* < 0.05).

**Conclusions:**

Although it is complicated to distinguish between causes and consequences, these findings suggest a complex and multifaceted, association between ED and HRQoL domains. Thus, multiple factors need taking into account in the policy of EDs prevention, identifying all the components of well-being to focus and personalize healthy programs in adolescences.

## Abstract (summary)

Adolescence is a transitional period from childhood to adulthood during which important biological, cognitive and social changes are experienced. In this phase, characterized by increased psychological vulnerability, classic risk behavior can develop, including altered eating behavior leading to eating disorders (EDs). EDs represent a varied group of pathological conditions influenced by various individual factors, including emotional state, cognitive functioning, and social context, probably associated to physical, emotional, and social changes that in young people can lead to great instability. Our results shown that is complicated to distinguish between causes and consequences that cause EDs, suggesting a complex and multifaceted association between ED and psychological domains. This is important in order to develop preventive and early intervention programs in the schools and in other educational institutions, focusing on what are considered potential predictor of EDs.

## Introduction

Adolescence is a period of transition from childhood to adulthood in which people undergo to many changes in almost areas of their life, including biological dimensions, cognitive functions, and social relationships [1]. Although most adolescents overcome these challenges successfully, adolescence is a time of increased risk behaviour, so much so that more than a third of adult psychopathologies find their onset during adolescence [2]. The possibility that particular risk factors for some developing young people may represent normal experiences, while for others they are indicators of a fragility that becomes a pathology over time, makes understanding psychological development in this time window particularly important [2]. In adolescence, within the body-mind relationship and especially in body-focused disorders, eating disorders (EDs) represent a varied group of pathological conditions influenced by various individual factors, including emotional state, cognitive functioning, and social context, probably associated to physical, emotional, and social changes that in young people can lead to great instability [3–7]. Despite the heterogeneity of the clinical and psychological condition, according the American Psychiatric Association, the three most common EDs are anorexia nervosa, bulimia nervosa, and binge eating disorder [8]. Currently epidemiological data indicate an increase in EDs, with a higher prevalence in female population as compared to males and frequent psychiatric comorbidities [9–12]. One of the most significant indicators is self-esteem that usually correlates with health and well-being [13–15]. In particular, positive self-esteem promotes better social adaptation and reduces the occurrence of risk behaviour [16]. Especially in adolescence, self-esteem is related to body image, more properly to body appreciation, considered a mediating factor for EDs. Body appreciation is a psychophysical construct that, when positive, is relates to healthy eating behaviour and good social relations, while is negatively associated with dysfunctional behaviors such as alcohol and drug use [17]. This gives insight into the complexity of EDs, but at the same time opens up the need to assess how EDs are associated with different health-related variables, not only the more purely physical ones, but above all those of an emotional and social nature. Among these variables impacting on EDs is certainly the phenomenon of dropping out of school, which to our knowledge has been little studied in acute cases, but is correlated in adulthood with a series of dysfunctional behaviours. Considering the impact of dropout on long-term health-related variables, the aim of this study was to assess the relationship between several dimensions of self-reported health-quality of life (HRQoL) and well-being perception with EDs in a sample of Italian school-dropout adolescents, with focus on possible sex differences.

## Materials and methods

### Participants

Data from school dropout were collected as part of AVATAR project, acronym for “A new purpose for promotion and eVAluation of healTh and well-being Among healthy teenageRs”. AVATAR project is aimed to develop a new tool to assess lifestyle habits, social context, and emotional status, in adolescents, and to define an integrated index of the best indicators of well-being [18]. In total, 680 boys and girls, aged between 18 and 21 years, were included. Adolescents who dropped out of school were enrolled in collaboration with the regional agency FORMATICA to which they were enrolled after dropping out for a professional training. School dropout adolescents were referred to the Tuscany Region’s FORMATICA Agency for a 2-year professional training course. This agency deals precisely with recovering those adolescents who have voluntarily interrupted their studies, offering them a training opportunity for a better integration into the professional world. The inclusion criteria were the following: absence of neuropsychiatric or other pathologies, signed informed consent and full completion of the proposed questionnaires.

Of 680 adolescents initially considered, 230 were excluded for the following reasons: diagnosed neuropsychiatric or other diseases (n = 20), absence of sign informed consent (n = 75), questionnaires not filled completely (n = 135). Therefore, the final population consisted of 450 adolescents (19 ± 2 years, male 308). Participants were previously instructed on how to fill out the questionnaires and to conduct the tests. One or two project members visited professional training course to provide the adolescents with verbal and written information about the data collection. Written information was also distributed to the parents. Active informed consent was obtained from adolescents. All tests were performed during participants’ computer lesson in school time. No incentive was provided to adolescents or parents. A research assistant was available to provide information and technical support to complete questionnaires.

### Ethical statement

The research protocol was approved by the local ethics committee review board. All subjects or legal guardians gave informed consent, and authorized physicians to use their clinical data in accordance with the Italian law. All procedures performed in the study were in accordance with the ethical standards of the institutional and/or national research committee and with the 1964 Helsinki declaration and its later amendments or comparable ethical standards (Regional Ethics Committee for Clinical Trials of the Tuscany Region 36/2021).

### Data collection

Data were collected with AVATAR Web-tool [18]. A socio-demographic data record was used to collect information on gender, age, and schooling. The Italian version of KIDSCREEN-52 was used to assess health-related quality of life [19, 20]. The KIDSCREEN is a self-report questionnaire designed to address health-related quality of life (HRQoL), aimed to monitor and measure the personal experiences in children and adolescent about their perception of health status and well-being. The questionnaire, that describes physical, psychological, mental, social, and functional aspects of well-being, consists of 52 items grouped in 10 dimensions (Physical well-being, Psychological wellbeing, Moods and emotions, Self-perception, Autonomy, Parent relations and home life, Social support and peers, School environment, Social acceptance (bullying), Financial resources) [19, 20]. Some sample items “In general, how would you say your health is?” for Physical well-being dimension; “Have you felt satisfied with your life?” for Moods and emotions; “Have you been happy with the way you are?” for Self-perception. Cronbach’s alphas are ranging from 0.77 to 0.89 for the dimensions of the 52-item version. KIDSCREEN questionnaires is psychometrically tested using data obtained in a multicentre European study which included a sample of 22,827 children recruited in 13 countries [21]. Except for mood and bullying, higher values of the variables express a better health-related quality of life. KIDSCREEN questionnaires is psychometrically tested using data obtained in a multicentre European study which included a sample of 22,827 children recruited in 13 countries. Eating attitudes and behaviours were assessed using EAT-26 consisting of three subscales: Dieting, eating preoccupation and oral control [22]. The EAT-26 consists of three sections: (a) self-reported height and weight to create a body mass index (BMI), (b) 26 items rated on a six-point Likert scale related to how often an individual engages in certain behaviors (“Always,” “Usually,” “Often,” “Sometimes,” “Rarely,” and “Never”), and (c) five behavioural items on a six-point Likert scale examining how often a person has engaged in disordered eating behaviors over the past 6 months (“Never,” “Once a month or less,” “2–3 times a month,” “Once a week,” “2–6 times a week,” and “Once a day or more”).

### Personalized well-being index

The AVATAR approach consists in focusing on the integration of three components of health-related well-being [lifestyle habits (LH); emotional status (ES); and social context (SC)], as perceived by adolescent [23]. The three components were obtained from the different variables analyzed by the questionnaires according to a structural model previously described in Mastorci et al. [24].

In detail, the path analysis technique used measures the extent to which the model fits a data set and allows testing of interrelationships between several variables simultaneously. The confirmatory factor analysis was used to test an overall measurement model that included five correlated latent variables. Overall model fit was assessed using different statistics. First, a chi-square analysis was used. The other indices were the root mean square error of approximation (RMSEA) (values between 0.05 and 0.08 indicate acceptable fit, and values < 0.05 a good fit), comparative fit index (CFI) (values > 0.90 indicate reasonable fit, > 0.95 good fit), and standardized root mean square residual (SRMR) (values < 0.10 indicate good fit). The measurement model was first tested to ensure that each of the observed variables was a sufficient indicator of the hypothesized latent variables.

From the sum of the three components, we obtained a personalized well-being index (PWBI), ranging from 0 to 100, according to the AVATAR model as reported before [24].

### Statistical analysis

Statistical data analyses were performed using SPSS software. Data are presented as mean ± SD, while categorical variables were presented as counts and percentages. The Saphiro–Wilk test was used to confirm the normality of data distribution for continuous variables before parametric analyses. A *p* value ≤ 0.05 was considered statistically significant.

One-way between-groups multivariate analyses of variance were performed to identify if EDs are associated with the dimensions of health-related quality of life and PWBI. These multivariate analyses were based on Wilks’ Lambda statistic after Bonferroni adjustment of significance for multiple tests based on 14 risk behaviors tested and on gender. In case of significance by the multivariate analysis, post-hoc analyses were conducted to explore inter-group differences for each dimension of health-related quality of life and PWBI.

## Results

### Association between eating disorders, health-related quality of life and personalized well-being index in study population and by gender

In total, 450 participants (32% girls, mean age 19.38 ± 1.89) were included in the analyses. Age was similar between male and female (male 19.35 ± 1.69 vs female 19.46 ± 2.28, *p* = ns).

Scores higher for risk of EDs were observed in the female population as compare to counterparts (14% vs 5.8%, *p* < 0.01). In total population, after adjustment for multiple tests, eating disorders was significantly associated with HRQoL as total score (Wilks' Lambda = 0.87, adj. *p* < 0.001). In particular, considering the single components of HRQoL, EDs were related to an impairment in physical (F = 9.289; *p* = 0.025) and psychological well-being perception (F = 26.964; *p* < 0.001), mood (F = 23.079; *p* < 0.001), and self-perception (F = 18.927; *p* < 0.001). After adjustment of HRQoL by gender, association between EDs and quality of life is maintained indicating that it is not due to gender (F = 14.220; *p* < 0.001).

With respect to well-being perception, assessed through PWBI, after adjustment for multiple tests, eating disorders in total population were significantly associated with PWBI (Wilks' Lambda = 0.89, adj. *p* < 0.001), in terms of reduction of score (F = 22.85; *p* < 0.001). Taking into account the three components of the index, EDs correlated with greater criticality of emotional status (F = 39.64; *p* < 0.001). After adjustment of PWBI by gender, association between EDs and well-being perception is preserved showing that it is not due to gender (F = 15.08; *p* < 0.001).

## Discussion

The first objective of this study was to identify the relationship between health-quality of life in its dimensions and well-being perception in its components with eating disorders perception in a sample of Italian school-dropout adolescents. Findings indicate an overall association between eating disorders and health-related quality of life and well-being perception in terms of total score. More specifically the presence of EDs is related to an impairment of physical and psychological well-being, mood and self-perception. The second objective was to analyse whether these relationships were sex-dependent, indicating that although there was a higher prevalence of EDs in the female population, this difference in the relationship with the dimensions related to well-being was not maintained. In other words, both in male and female adolescents, EDs negatively impacts on psychosocial dimensions.

Eating disorders are a category of disease often under-diagnosed and many adolescents go untreated, do not recover or recover only partially. High rates occur in younger children, adolescents and minority groups, presenting themselves differently in these populations, which is why recognizing them early is not always easy, although to our knowledge there is no data on EDs among our study sample, school-dropout adolescents [7]. In line with previous data, a relationship between self-perception and EDs was shown [25]. Our results are consistent with that reported by prior research, revealing the presence of EDs when self-perception is low [26]. Of all health-related dimensions, in facts, self-perception in adolescence appears to be the most important, even playing the role of protective or preventive factor against EDs [27]. Indeed, it is common that low self-perception co-occurs with other psychological traits of EDs creating a vicious cycle. On the other hand, our results demonstrated also a relation with an impairment in physical and psychological well-being perception and reduced mood level, suggesting a compromised emotional profile. Generally, during adolescence, these constructs may be associated with the definition of one's body image. Traditionally, body image and health are considered associated only in pathological conditions, mainly because a negative body perception is thought as a risk factor for eating disorders and other psychological diseases.

In this frame, as suggested by our results, there is another important aspect concerning the association between the perception of body size and mental health, and subsequent impact on EDs [28]. In a previous study, our group demonstrated that, in adolescent, exists a closed relationship between weight status categories and HRQoL, especially in girls and particularly in underweight girls, who presented a greater perception of well-being, pointing out how this in the long term can contribute to psychological distress that can lead to an eating disorder [29]. This is in line with our notion that young female usually reports aim to weight loss, whereas a higher percentage of boys report the goal to maintain their weight. With regard to sex differences and the possible different association between EDs, HRQoL, and PWBI, our results have shown that these relationships are present equally both in male and in female students, although the prevalence of EDs was higher in girls as expression of a biological predisposition, although female population is composed only by 32% [30]. These findings are probably due to the fact that EDs are affected by cultural standards related to the body as a social fact rather than a biological one [31]. Usually, the predominance of research on eating disorders, also in terms of prevention, focuses mainly on females, although in recent years the percentage of males with these disorders has increased significantly, with onset beginning at around age 11 [32]. Although also the data on the co-occurring psychological conditions are more described in females, some studies reported male with EDs more likely to have depression, even if they are still inconsistent data [33]. In general, the inconsistency of data on possible sex differences and related co-morbidities that may somehow exacerbate EDs, may be due on the different weight control behaviors; boys are potentially focusing on developing muscularity, while females on leanness [34]. In fact, in the male gender, factors such as body image related to muscularity and sexual orientation may increase the risk of developing eating disorders [35]. In young women, on the other hand, the beauty ideals proposed by social media seem to have a great influence. Indeed, there is a relationship between body image, body concerns, body dissatisfaction and disordered eating attitudes in the female gender [36]. In particular, in girls has been described an association between time spent on social networks and more significant eating disorders symptoms [37].

Notable possible strengths of this study include the possibility to develop preventive strategies on psychosocial dimensions in order to reduce the vulnerability and the incidence of the ever-increasing EDs in adolescent. In addition, it is important to emphasize that this is a first pilot study conducted on school-dropout adolescents, who are therefore potentially more exposed to risk factors, as the little literature aimed at this adolescent population group indicates. A recent work of our group revealed that school-dropout girls are characterized by a compromised health indicator profile in terms of both risk behaviors, including EDs [38]. The study of the dropout phenomenon is new, as most of the evidence focuses on the long-term effects of dropping out of school, but there are no studies to our knowledge on the short- and medium-term effects. School dropout opens up the possibility of developing risky behavior and vulnerability to disease in adulthood. So, it is like having an increased susceptibility, in a biological period already of increased fragility as adolescence.

Although the current study provides the groundwork for future research, certain limitations warrant discussion. First, the sample consisted of 450 dropout adolescents between the ages of 18 and 21 and identified as predominately of North Italy, are not representative of overall Italian Country neither of school-dropout students. Also, the relatively small sample size may have limited power to detect smaller effect sizes, especially as it relates to EDs, HRQoL categories, and PWBI. Future studies on a larger and more representative population will be necessary to confirm our preliminary results. Furthermore, in light of the results of our previous research on the link between body weight and quality of life, it would also have been important to include body weight. However, this was not done as it was self-assessed and may not be a real result. Considering the role of social networks in the onset of EDs, in future studies on the relationship between mental functioning and development of body-focused disorders, it will also be important to consider this aspect. Furthermore, a more accurate association between HRQoL and EDs could be studied using the recently validated Italian version of the Eating Disorder Quality of Life [39]. Another important aspect to assess is sexual orientations, because, especially in the analysed age, has a serious role in the EDs onset and psychopathology.

Lastly, more important information about EDs and psychosocial profile would be obtained if they compared those who dropped out to those who did not drop out.

## Conclusion

The current study demonstrates that there is an association between EDs and health-related quality of life and well-being perception; in other terms when the disorder is present are impaired physical and psychological well-being, mood and self-perception. However, males and females reported the same level of these associations, in alignment with some previous reports. Long-term monitoring is needed to evaluate whether these impairments persist over time and how they affect the psychosocial sphere in terms of co-morbidity. Our results are important because they refer to an at-risk cohort although obtained on a small pilot sample, they shed light on a complex and multifaceted phenomenon such as school-dropout. In this sense, it is as if in our sample coexisted psychological, physical, and social alterations typical of EDs and the same changes characteristic of school-dropout, without however knowing who causes what. Future research elucidating the nature of EDs in school-dropout adolescents, and how the alterations here presented may differ from regular students, is warranted. Additional research establishing the psychosocial features of EDs symptom in school-dropout adolescents is also required, in order to develop preventive and or early intervention programs in eating disorders. Promoting health has long been a pivotal role of schools and of other educational institutions, but traditionally these activities have focused mainly on general health education. In this context, according our results, it would be useful to focus on self-esteem, which not surprisingly is impaired in school-dropout and is reputed to be a predictor of EDs.

## Data Availability

The datasets used and/or analyzed during the current study are available from the corresponding author on reasonable request.

## Abbreviations

ED: Eating disorders
HRQoL: Health-related quality of life
WBP: Well-being perception
AVATAR: A new purpose for promotion and eVAluation of healTh and well-being Among healthy teenageRs
LH: Lifestyle habits
ES: Emotional status
SC: Social context
